# Macroscopic modeling of mammalian cell growth and metabolism

**DOI:** 10.1007/s00253-015-6743-6

**Published:** 2015-07-22

**Authors:** Bassem Ben Yahia, Laetitia Malphettes, Elmar Heinzle

**Affiliations:** Biochemical Engineering Institute, Saarland University, Campus A1.5, D-66123 Saarbruecken, Germany; Upstream Process Sciences Biotech Sciences, UCB Pharma S.A., Avenue de l’Industrie, B-1420, Braine l’Alleud, Belgium

**Keywords:** Cell culture, Macroscopic modeling, Antibody production, Kinetic models, Stoichiometric relationships, Quality by Design

## Abstract

We review major modeling strategies and methods to understand and simulate the macroscopic behavior of mammalian cells. These strategies comprise two important steps: the first step is to identify stoichiometric relationships for the cultured cells connecting the extracellular inputs and outputs. In a second step, macroscopic kinetic models are introduced. These relationships together with bioreactor and metabolite balances provide a complete description of a system in the form of a set of differential equations. These can be used for the simulation of cell culture performance and further for optimization of production.

## Introduction

Mammalian cell cultures are the major source of a number of biopharmaceutical products, including monoclonal antibodies (Niklas and Heinzle 2012; Sidoli et al. 2004), viral vaccines (Vester et al. 2010), and hormones (Nottorf et al. 2007). Chinese hamster ovary (CHO) cells are widely used as an expression system for the synthesis of therapeutic glycosylated proteins (Palomares et al. 2004; Zhu 2012). Predicting the behavior of mammalian cells during cell culture processes under different culture conditions is highly desirable for both commercial and scientific reasons (Kell and Knowles 2006). In batch and fed-batch processes, the rate of overproduction of heterogeneous proteins by mammalian cells is limited by the decline in cell viability, by the depletion of required metabolites and substrates or by the accumulation of metabolic products and inhibitors. Therefore, it becomes imperative to identify the parameters which have a significant impact on cell viability and on protein production and understand their effects on the cellular phenotype. Moreover, in 2004, the Food and Drug Administration (FDA) proposed the “Quality by Design” (QbD) methodology to biopharmaceutical companies. The focus of this concept is that the quality, most important protein glycosylation, should be built into a product with a thorough understanding of the product itself and the process for its production (Tomba et al. 2013). Additionally, critical process parameters should be identified which have an impact on the critical quality attributes (CQAs) of the product (Kontoravdi et al. 2007; Royle et al. 2013; Teixeira et al. 2009).

Mammalian cell culture processes are complex (Stelling et al. 2006), and numerous input parameters have to be identified to optimize growth and productivity (Nolan and Lee 2011, 2012; Sellick et al. 2011). To understand biological mechanisms and to optimize production processes, rational design guided by experience is the most common method currently used. However, experiments are time consuming and expensive to perform and, generally, generate noisy data. Mathematical models can help to characterize the different phenotypes and the needs of mammalian cells (Royle et al. 2013; Sidoli et al. 2004). They can be used as a prediction tool in simulation and optimization (Goudar et al. 2006; Wiechert 2002). Mathematical models can also help to understand and identify mechanisms that cannot be easily identified only with experimental data and a pure statistical analysis of them. Therefore, modeling of metabolism has become highly desirable in the development process where the identification of the parameters impacting the cell culture processes and the prediction of the evolution of the processes are important. Identification of yield coefficients can be used for this purpose (Chen and Bastin 1996). This creates significant added value in terms of cost and time compared to methods that do not use models (Kessel 2011).

Compared to very detailed cellular models, the benefit of the use of macroscopic models is that it is much easier but yet very informative to analyze the cells as a black box or grey box rather than to take into account extended details of what happens inside the cell (Zamorano et al. 2013). Analysis of intracellular metabolites necessary for setting up and tuning detailed kinetic models of metabolism is much more complex to perform than extracellular metabolite analysis and requires much more sophisticated techniques, particularly for suspended cells (Neermann and Wagner 1996; Wahrheit and Heinzle 2013). In addition, the number of model parameters in macroscopic models is significantly lower than the number of parameters in microscopic models. The identification of parameters is therefore more difficult for very detailed microscopic models.

A mathematical model can be used for different purposes (Ashyraliyev et al. 2009; Hu 2012):

1. To summarize a large volume of experimental data,
2. To explore concepts and test hypotheses,
3. To predict the behavior of the systems under non-tested conditions,
4. To identify conditions for optimal performance of a process as defined by an objective function.

The extrapolation power of a model cannot be predicted a priori. The probability that a model will allow prediction outside the originally observed region is, however, increasing if physically meaningful functions are used. In our review, we emphasize the separation into a material balancing part, the so-called macroscopic reactions, and a kinetic part. The material balancing part, i.e., stoichiometry, provides a sound basis and must not be violated for keeping predictivity. The kinetic part relies very much on the characteristics of the rate determining processes, e.g., saturation kinetics of Michaelis-Menten type, allosteric kinetics of Hill-type, or structure of feedback control loops in biological systems. The appropriate choice of the underlying types of mathematical functions is certainly a crucial point in this respect. For certain problems, e.g., metabolic network modeling as shown for CHO, the use of ensembles, i.e., sets of models with different structures and/or parameter values, seems useful for improving prediction (Villaverde et al. 2015).

In this review, we will present different types of models used in previous work to model the metabolism of suspension cells at the macroscopic level, i.e., to model extracellular outputs as function of extracellular inputs. This paper is organized as follows: (i) the first part introduces the types of models and the existing modeling frameworks (Mahadevan and Doyle 2003). (ii) Then, different methods for identifying relevant parameters for creating a macroscopic metabolic model will be presented. Preliminary work has to be performed to reduce the number of parameters to study and to understand which parameters have a significant impact on the responses (Mahadevan and Doyle 2003). (iii) In part three, different kinetic models are reviewed. Kinetic models are used after the selection of parameters and when the relationships between those parameters are defined. (iv) Model calibration and testing are reviewed and (v) applications to process control are described. (vi) Finally, main conclusions and an outlook are presented.

## Types of models

There are different ways to classify models. The first distinguishes between empirical models, also called descriptive models, and mechanistic models. Empirical models use a pragmatic description of all the data with any suitable mathematical relationship. They only partially take into account the underlying phenomena or physical laws that govern the system behavior. Mechanistic models are based on theoretical foundations of systems and on known relationships. The predictions of the responses are based on biological, chemical, and physical input of knowledge.

Another classification was proposed by Tsuchiya et al. (1966) and distinguishes deterministic models and probabilistic models. The first is based on continuous variables using differential equations. Reactions and interactions are represented as continuous processes (production, consumption, growth…) by corresponding mathematical functions. It is appropriate for systems composed of a relatively large number of cells, e.g., more than 10,000. This kind of model describes the population as average. Probabilistic or stochastic models use probability in the formulation of the model and are typically used for a population of only few cells or for molecular events with only small number of molecules, e.g., transcription. This allows representation of the variability of a population and a system. In cell culture, the number of cells is usually very large (e.g., >10^6^ cells/ml) allowing the preferential use of deterministic models.

Another classification distinguishes structured, non-structured, segregated, and non-segregated models. Structured models take into account the cellular reactions within cells (Harder and Roels 1982; Tsuchiya et al. 1966). Structured models can describe biological systems in great detail but are more difficult to set up. The number of parameters increases with the complexity of the model and with the number of intracellular reactions taken into account. In addition, despite the enormously increased knowledge about cellular process, there is still a significant lack of information about many steps, e.g., transport, control of enzymes activities and expression or post-transcriptional processing of proteins. Unstructured models are easier to work with because they analyze the cells as a black or grey box. Intracellular reactions are not analyzed in detail. It is assumed, for example, that cell growth depends only on extracellular parameters. However, the extended and now easily excessible comprehensive knowledge about the biochemical reaction networks and its stoichiometry allows the incorporation of this information into macroscopic models. Macroscopic models are less accurate than structured models, but easier to set up and to apply. Segregated models, as opposite of non-segregated models, describe cellular behavior as a function of cell cycles or age of cells (García Münzer et al. 2015a, b; Karra et al. 2010; Meshram et al. 2013; Pisu et al. 2015). The vast majority of models are non-structured and non-segregated.

Neural networks are particularly useful to relate input and output variables to each other in complex systems with incomplete or even completely lacking knowledge of the systems structure and also in cases with incomplete measurements. Mechanistic knowledge can however be introduced by using hybrid models (Oliveira 2004; van Can et al. 1999).

## Identification of relevant input-output relationship

A general macroscopic reaction scheme of macroscopic reactions can be expressed as follows (Bastin and Dochain 1990):

1 $$ {\displaystyle \sum_{i\in {R}_k}\left(-{\nu}_{i,k}\right)}{\xi}_i\overset{\varphi_k}{\to }{\displaystyle \sum_{j\in {P}_k}{\nu}_{j,k}}{\xi}_jk\in \left[1,M\right] $$

where

- M is the number of reactions;
- ${\varphi}_k$ is the *k*th reaction rate;
- ${\xi}_i$ and ${\xi}_j$ are the *i*th and the *j*th component, respectively;
- ${\nu}_{i,k}$ and ${\nu}_{j,k}$ are the corresponding stoichiometric coefficients;
- ${R}_k$ is the *k*th set of reactant and catalyst indices;
- ${P}_k$ is the *k*th set of product and catalyst indices.

**Fig. 1:**
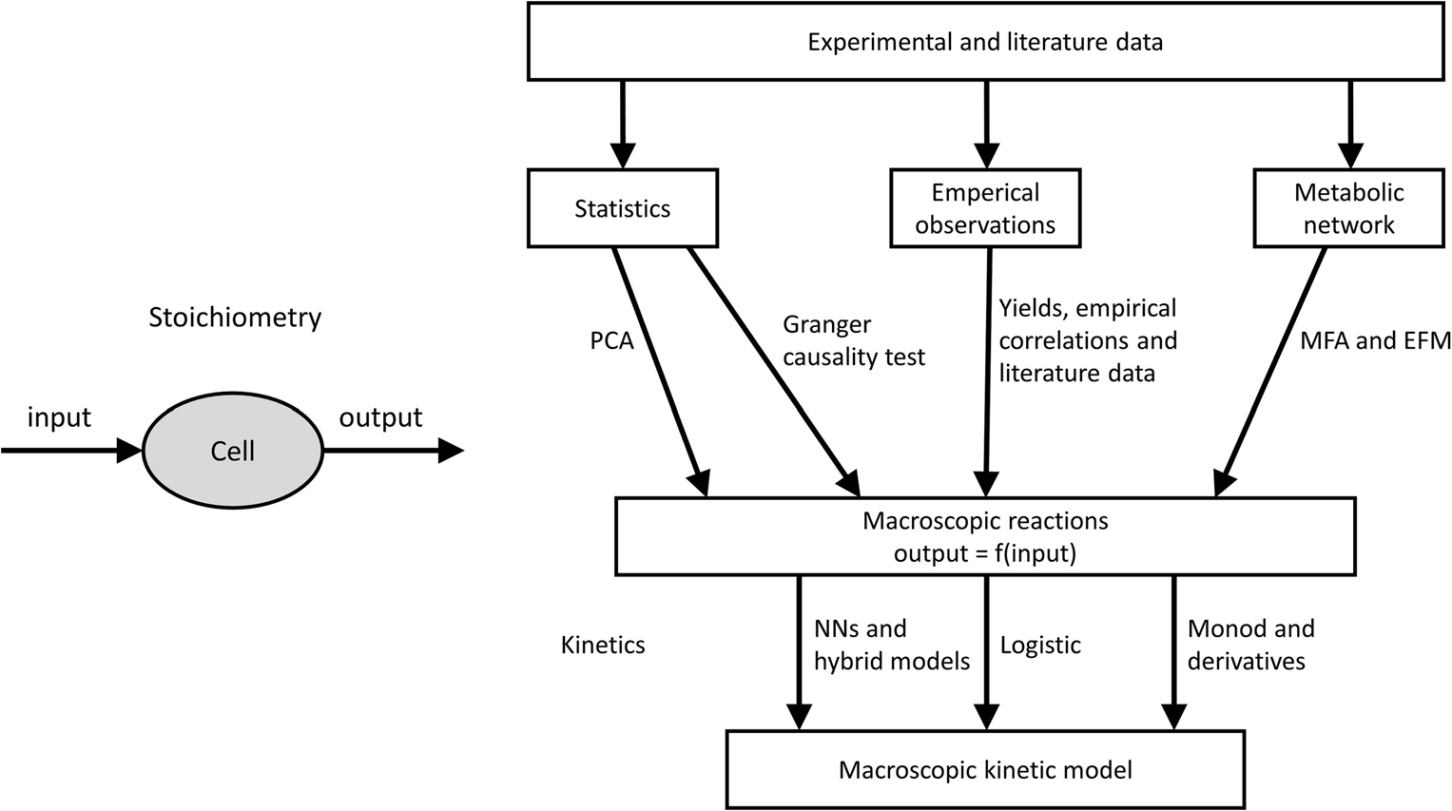
Methods to derive macroscopic kinetic models. In order to get a simulation and prediction model of the macroscopic cell behavior, first, the macroscopic reactions of the cell culture system have to be determined, i.e., the stoichiometry relating input and output of the cells. To do that, statistical methods, empirical observations, and metabolic network-based methods can be used. After that, the kinetics of the system have to be described and combined with the stoichiometric model. Finally the model is calibrated, usually using optimization-based methods, and tested. *PCA* principal component analysis, *MFA* metabolic flux analysis, *EFM* elementary flux mode, *NNs* neural networks

This general reaction scheme represents a macroscopic stoichiometric relationship. To set up such a macroscopic model, the important parameters, i.e., the relevant cellular inputs, ${\xi}_i,$ and outputs, ${\xi}_j,$ as well as the stoichiometric coefficients, ${\nu}_{i,k},{\nu}_{j,k},$relating the inputs to the outputs, have to be determined. This can start from the increasingly comprehensive knowledge of cellular reactions and transport or, as traditionally done, from purely empirical data. Ideally, both types of information are combined as described below and indicated in Fig. 1. This step is often the main bottleneck in the design of a macroscopic model for complex biotechnological processes.

### Method based on expert reasoning

One possible approach to select significant parameters is based on expert reasoning and experimental observations. This approach measures correlations between the macroscopic outputs we want to model with the cell culture parameters, i.e., the macroscopic inputs, under different experimental conditions. A most popular method uses the concept of yield coefficients relating always two measured variables to each other, e.g., biomass to substrate or product to biomass (Dunn et al. 2003). Yield coefficients are frequently used to set up stoichiometric relationships to be applied in metabolic flux analysis using metabolite balancing (Niklas et al. 2009). It requires little thought about the actual detail of the system and uses most significant phenomena observed during experiments to define the extracellular parameters such as limiting nutrients or accumulation of side waste products. Typically, outputs/inputs taking into account in a macroscopic model with this kind of approach are biomass, glucose, glutamine, lactate, and ammonia. For instance, Jang and Barford (2000) developed an unstructured model of growth and metabolism of a mouse murine hybridoma AFP-27 cell line producing an IgG1 antibody. They assumed that glucose, glutamine, lactate, and ammonia were growth limiting. Lactate and ammonia were considered as toxic products of catabolic reactions, which inhibit cell growth and can ultimately cause cell death in their model; even though they assumed that hybridoma cells can produce monoclonal antibodies until any of amino acid is depleted, they only considered glutamine as a limiting amino acid. Moreover, based on the demonstration of Suzuki and Ollis (1990), they considered the specific antibody production to be a function of the fraction of cells in G1 phases. Acosta et al. (2007) also assumed this link between specific growth rate and specific productivity in their model of IgG2a Mab production in hybridoma cells. Although glucose is generally important for cell growth, it was not found to be a limiting nutrient in another model (Bree et al. 1988) that is, however, only relying on one batch experiment, certainly a too limited observed experimental space for meaningful extrapolation. Lactate and ammonia are assumed to both inhibit and kill cells (Batt and Kompala 1989; Glacken et al. 1988; Ozturk et al. 1992), but the impact on specific antibody productivity was reported as not significant (Ozturk et al. 1992). Jang and Bradford (2000) and Dhir et al. (2000) assumed that the lactate production was due to cellular consumption of glucose and glutamine. They assumed that the spontaneous degradation of glutamine was negligible. It is, however, usually relevant but depending on the used medium and process duration (Borchers et al. 2013; Glacken et al. 1988; Ozturk and Palsson 1990). Amino acid depletion has been considered in another model developed by Liu et al. (2008). Knowledge about metabolism and its control can be incorporated but usually not in a systematic manner. Meshram et al. (2013) developed a macroscopic metabolic model and linked it to a model of apoptosis. A dynamic model of Mab synthesis and Mab glycosylation by hybridoma was described by Kontoravdi et al. (2007) using a structured model based on the work of Umaña and Bailey (1997). The availability of nutrients such as glucose or glutamine had an impact on protein glycosylation.

Such empirical procedures can be a valuable tool for understanding metabolic processes as well as for process design and optimization. They are used to design a macroscopic model and select the extracellular parameters which have an impact on the response defined. Nevertheless, very little real understanding of the cell culture process is obtained with this kind of procedure.

### Method based on statistical tools

A large number of variables can be identified and quantified due to the recent development of high-resolution and high-throughput analytical techniques (Martin et al. 2014; Steinhoff et al. 2014). In this context, it becomes more complex to select the significant input only with an empirical approach and based on expert judgment. Moreover, the relations of variables are generally dynamic and involve temporal dependencies.

To deal with these challenges, multivariate data analysis methods, e.g., principal component analysis (PCA), can be used as a statistical tool to select parameters. PCA is a multivariate analysis method based on eigenvalue analysis, which is actually the projection of original data onto a new set of axes, i.e., the principal components. PCA has been introduced by Pearson (1901) and Hotelling (1933) to describe the variation of multivariate data in terms of a set of uncorrelated variables. It is used to reduce a high-dimensional dataset into fewer dimensions while retaining important information. Starting out with high-dimensional noisy experimental data, one can reduce the dimensionality and even remove pure random errors by determination of significant factors (Malinowski 1991). Using significant factor analysis followed by rotation, a stoichiometric model with only two independent, physically meaningful reactions were identified for *Bacillus subtilis* batch culture (Saner et al. 1992). Xing et al. (2008) used a methodology based on principal factor analysis (PFA) to identify threshold values of repressing metabolites, i.e., ammonium, lactate, osmolality, and carbon dioxide levels, on CHO growth and protein quality (glycosylation properties) but also to select significant inputs. PFA was applied by rotating principal components obtained by PCA and seeks physically meaningful linear combinations of variables. In their study, Xing et al. determined that ammonia and glucose negatively contributed to cell growth. Lactate and osmolality were positively correlated to cell growth and pCO_2_ levels can reduce protein quality above a defined threshold. Multivariate analysis methods can be a powerful tool to determine the macroscopic stoichiometry of a biological system that cannot easily be determined by intuition. However, it becomes more complex to evaluate correlations and to apply this kind of statistical method with time-series data with varying number of metabolic phases, particularly in fed-batch cultures.

Another possibility to deal with this complexity is to use time-series data analysis such as the Granger causality test. The Granger causality test is a statistical hypothesis test used to determine causality among parameters. It was developed by Clive Granger (1934–2009), a British economist (Granger 1969). This test has recently been used to analyze transcriptomics and metabolomics profiles (Sriyudthsak et al. 2013). Siryudthsak et al. introduced this test to evaluate causality among metabolites. Direct relationships between two metabolites were evaluated using the bivariate Granger causality test. This method has not yet been used to develop macroscopic metabolic reactions and to select the significant input parameter, but it is expected to be applied in the future.

Statistical tools are useful when the underlying phenomena are too complex to resolve manually, such as multivariate data or temporal data. The two statistical methods presented above can help to structure problems, to reduce the dimensionality of the problem, to select relevant input and output parameters, and to develop a macroscopic stoichiometric model.

### Method based on metabolic network knowledge

The central idea is that the macroscopic behavior of cellular metabolism is the result of a combination of intracellular microscopic reactions that are more and more easily accessible via public databases. Metabolic networks are represented as a system of metabolite balance equations based on stoichiometric reactions. The general goal is to identify a minimal set of macroscopic reactions that can then build a sound basis for a macroscopic model.

#### Network construction

Metabolic network models of the central metabolism of mammalian cells have been built from the available genomic and biochemical information. Multiple databases can be used as resource for metabolic network reconstruction. As an example, the Kyoto Encyclopedia of Genes and Genomes (KEGG) pathway database (Kanehisa et al. 2014) and the BioCyc database collection (Caspi et al. 2014) are important databases that can be used to reconstruct a metabolic network. A number of studies have proposed metabolic networks of central metabolisms (Ahn and Antoniewicz 2012; Antoniewicz 2013; Nicolae et al. 2014; Zamorano et al. 2013). To set up stoichiometric macroscopic relationships of cell metabolism, the main difficulty is the size of the metabolic network which can make the decomposition into external macroscopic reactions complex (Rügen et al. 2012). To overcome this problem, metabolic networks can be reduced and simplified using computed fluxes in order to detect and remove insignificant pathways.

#### Metabolic flux analysis

Metabolic flux analysis (MFA) using metabolite balancing, first applied for microorganisms (Aiba and Matsuoka 1978), has been widely applied to mammalian cells. Metabolite balancing is a powerful method to quantify the manifestation of a phenotype (Ahn and Antoniewicz 2012; Antoniewicz 2013; Goudar et al. 2006, 2009; Grafahrend-Belau et al. 2013; Klein et al. 2013; Niklas and Heinzle 2012; Niklas et al. 2011; Quek et al. 2010; Sengupta et al. 2011; Varma and Palsson 1994; Wahrheit et al. 2014b). Metabolite balancing is based on the assumption that accumulation of intracellular metabolites is insignificant compared to the extracellular fluxes in batch and fed-batch cultures (Niklas and Heinzle 2012). This assumption is valid for small concentration of intracellular metabolites which is usually fulfilled but may deviate to a certain extent for highly concentrated metabolites, e.g., of the TCA cycle (Rehberg et al. 2014). Based on this quasi-steady-state assumption, we can say that the sum of influxes and effluxes of an internal metabolite of a metabolic network is equal to zero.

2 $$ S\cdotp v=0 $$

where S is a stoichiometric matrix, based on a defined metabolic network, with each row corresponding to a balanced internal metabolite and each column corresponding to a flux in the flux vector, *v*.

We can then split Eq. 2 to have on one side, the fluxes that are experimentally measured (substrates, products, biomass), *v*_*m*_, and on the other side, fluxes that will be calculated by MFA, *v*_*c*_:

3 $$ {S}_m\cdotp {v}_m=-{S}_c\cdotp {v}_c $$

*S*_*m*_ and *S*_*c*_ are the stoichiometric matrices associated to *v*_*m*_ and *v*_*c*_, respectively. If *S*_*c*_ is a square matrix of full rank, the fluxes are calculated by

4 $$ {v}_c=-{\left({S}_c\right)}^{-1}\cdotp {S}_m\cdotp {v}_m $$

The uptake and production rates of metabolites are such measurable external fluxes that can be related to the specific growth rate, *μ*, by yield coefficients ${Y}_{\mathrm{Met}/Bio}$.

5 $$ {v}_{m,i}=\mu \cdotp {Y}_{\mathrm{Met}/Bio} $$

Monte Carlo simulation can be used to get a more precise and realistic estimation of the standard deviation of the calculated fluxes. A dynamic metabolic flux analysis can also be performed in order to have the profile of the intracellular flux over time (Niklas et al. 2011; Wahrheit et al. 2014a). When metabolite balancing is performed, reactions with insignificant fluxes can be identified and then deleted from the metabolic network to simplify it.

#### Elementary flux mode analysis

Elementary flux mode (EFM) analysis can then be applied on a metabolic network as defined in Eq. 2. EFM analysis is the calculation of independent, minimal biochemical pathways in a metabolic network at steady-state, which are thermodynamically and stoichiometrically possible, taking into account the irreversibility or the reversibility of the reactions (Schuster et al. 1999). There is a distinction between external and internal metabolites. A “flux mode” is a steady-state flux distribution in which the proportions of fluxes are fixed and it is called “elementary” if it is not decomposable. Various software can be used for this purpose such as COPASI (Hoops et al. 2006), Metatool (Schuster and Schuster 1993), efmtool (Terzer and Stelling 2008), or CellNetAnalyser (Klamt and von Kamp 2011).

To perform EFM, the stoichiometric matrix based on a metabolic network is used, and the convex basis vectors are computed using Eq. 2, taking into account the thermodynamic feasibility constraints (Schuster et al. 1999). Any possible flux distribution *v* can be expressed as a non-negative linear combination of a set of elementary flux vectors *e*_*i*_ which represent the not decomposable metabolic paths between the substrates and the final products.

6 $$ v={\omega}_1{e}_1+{\omega}_2{e}_2+\dots +{\omega}_p{e}_p\ {\omega}_i\ge 0 $$

The non-negative matrix *E* with column vectors *e*_*i*_ satisfies *S*·*E* = 0. *E* constitutes the admissible flux space also known as the convex polyhedral cone (Gagneur and Klamt 2004). However, a critical issue in EFM is the calculation of these elementary flux vectors because of dramatically increasing computational demands with increasing network size. Based on the matrix *E*, a set of macroscopic reactions of the extracellular metabolites can be derived (Baughman et al. 2010; Dorka et al. 2009; Gao et al. 2007; Niu et al. 2013; Provost and Bastin 2004; Provost et al. 2006; Zamorano et al. 2013). Examples of the stochiometric matrix *E* are presented in Table 1. A methodology was proposed by Junger et al. (2011) to compute minimal elementary decompositions of metabolic flux vectors. Later, Zamorano et al. (2013) showed that this method allows the estimation of metabolic fluxes even with an underdetermined mass balance system where data are not sufficient to uniquely define these fluxes. This provides also an excellent basis for setting up macroscopic models.

**Table 1:** Stoichiometric matrices of macroscopic reaction networks for CHO cell lines

	e1	e2a	e2b	e3	e4	e5	e6	e7	e8	e9	e10
Glucose	−**1**	−**1**	−**1**	−**1**	0	0	0	−**0**.**0508**	0	0	0
Gln	0	−**2**	0	0	0	0	−**1**	−**0**.**0577**	−**0**.**0104**	−**1**	0
Lac	**2**	0	**2**	**2**	0	0	0	0	0	0	0
Glu	0	**2**	−**2**	−**2**	−**1**	0	**1**	−**0**.**0016**	−**0**.**0107**	**1**	−**1**
Asn	0	0	0	0	0	−**1**	**1**	−**0**.**006**	−**0**.**0072**	0	0
Asp	0	0	0	0	0	**1**	−**1**	−**0**.**0201**	−**0**.**0082**	0	0
Ala	0	**2**	**2**	0	0	0	0	−**0**.**0133**	−**0**.**011**	0	0
Pro	0	0	0	0	**1**	0	0	−**0**.**008**	−**0**.**0148**	0	0
BM	0	0	0	0	0	0	0	**1**	0	0	0
Mab	0	0	0	0	0	0	0	0	**1**	0	0
(Dorka et al. 2009)											
	e1	e2	e3	e4	e5	e6	e7	e8	e9		
Glucose	−**1**	−**1**	−**1**	0	0	0	−**0.0508**	0	0		
Gln	0	0	0	0	0	−**1**	−**0.0577**	−**0.0104**	−**1**		
Lac	**2**	**2**	**2**	0	0	0	0	0	0		
NH_3_	0	0	0	0	**1**	0	0	0	**1**		
Glu	0	−**2**	−**2**	−**1**	0	**1**	−**0**.**0016**	−**0**.**0107**	**1**		
Asn	0	0	0	0	−**1**	**1**	−**0**.**006**	−**0**.**0072**	0		
Asp	0	0	**2**	0	**1**	−**1**	−**0**.**0201**	−**0**.**0082**	0		
Ala	0	**2**	0	0	0	0	−**0**.**0133**	−**0**.**011**	0		
Pro	0	0	0	**1**	0	0	−**0**.**0081**	−**0**.**0148**	0		
CO2	0	**2**	**6**	0	0	0	0	0	0		
BM	0	0	0	0	0	0	**1**	0	0		
Mab	0	0	0	0	0	0	0	**1**	0		
(Gao et al. 2007)											
	e1	e2	e3	e4	e5	e6	e7				
Glc	−**1**	−**1**	0	0	0	−**1**	−**1**				
Gln	0	0	−**1**	−**1**	−**1**	−**3**	−**2**				
Lac	**2**	0	0	1	0	0	0				
NH_3_	0	0	**1**	**2**	**2**	**1**	**1**				
Ala	0	0	**1**	0	0	0	0				
CO_2_	0	**6**	**2**	**2**	**5**	**2**	**2**				
Nucl	0	0	0	0	0	**1**	**1**				
(Provost and Bastin 2004)											
	e1	e2	e3	e4	e5	e6	e7	e8	e9		
Glc	−**1**	−**1**	−**1**	0	0	0	−**0**.**0508**	0	0		
Gln	0	0	0	0	0	−**1**	−**0**.**0577**	−**0**.**0104**	−**1**		
Lac	**2**	**2**	**2**	0	0	0	0	0	0		
NH_3_	0	0	0	0	**1**	0	0	0	**1**		
Glu	0	−**2**	−**2**	−**1**	0	**1**	−**0**.**0016**	−**0**.**0107**	**1**		
Asn	0	0	0	0	−**1**	**1**	−**0**.**006**	−**0**.**0072**	0		
Asp	0	0	**2**	0	**1**	−**1**	−**0**.**0201**	−**0**.**0082**	0		
Ala	0	0	0	**1**	0	0	−**0**.**0081**	−**0**.**0148**	0		
Pro	0	**2**	0	0	0	0	0	0	0		
BM	0	0	0	0	0	0	**1**	0	0		
Mab	0	0	0	0	0	0	0	**1**	0		
(Baughman et al. 2010)											

Numbers differing from 0 are given in bold

*Glc* glucose, *Lac* lactate, *BM* biomass, *Mab* monoclonal antibody, *Nucl* nucleotides, amino acids are specified using the standard three-letter code

The output of these approaches are stoichiometric macroscopic relationships of cell metabolism based on a metabolic network and on biological and biochemical knowledge that provide a necessary input for kinetic macroscopic models.

## Macroscopic kinetic models

After a first screening to select input parameters and to set up the stoichiometric macroscopic reactions, the macroscopic kinetic reactions can be developed. Different types of kinetics are available and this section will present some of the most important ones.

### Monod model and its derivatives

For modeling of mammalian cell culture kinetics, the Monod equation and derivations of it are most frequently applied. These kinetics with slight modifications are capable to simulate different types of characteristics like saturation, inhibition, and limitation by substrates and other components.

For Monod kinetics, the growth is defined as follows:

7 $$ \mu ={\mu}_{\max}\left[\prod \frac{S_i}{S_i+{K}_{Si}}\right] $$

Where *μ* is the specific growth rate; *S*_*i*_ and *K*_*Si*_ are the corresponding substrate concentration and half-saturation constant, respectively. *μ*_max_ is the maximum specific growth rate. To incorporate inhibitory effects, a corresponding term is added to the denominator. In the case of balanced growth, all other rates can be related to *μ* by yield coefficients (Eq. 5).

There are two methods for estimation of the maximum specific growth rate, *μ*_*max*_, and the associated Monod constant, *K*_*Si*_. One is the steady-state measurement of growth and the limiting substrate concentration in continuous culture at different dilution rates. An alternative method is the measurement of growth rate at different substrate concentrations in batch culture (Banerjee 1993). To estimate the maximum specific growth rate, *μ*_*max*_, the associated Monod constant, *K*_*Si*_, were arbitrarily set to small values to obtain balanced growth (Dorka et al. 2009; Provost et al. 2006). This seems well justified for batch cultures but will not allow to transfer such a model to continuous or fed-batch processes without readjustment of these constants. Monod-type models are widely used, but it is often difficult to define which formulation is the best to characterize the cell behavior (Bastin and Dochain 1990). Furthermore, finding the optimal formulation of this kind of model and estimating model parameters can be time consuming. Table 2 presents kinetic growth models used in the literature to describe growth of different organisms. Generally, only the growth rate is described by a Monod-type model, and the other components, products, and substrates are then described by simple mass balance equations (Baughman et al. 2010; Borchers et al. 2013; Sainz et al. 2003; Xing et al. 2010), also called first principle models (FPMs). To describe the relationship between the variation of substrates and products with the cell number, the mass balance equations are defined as a set of ordinary differential equations (ODEs) based on biological knowledge and taking into account the inner structure of the cells. Often the specific consumption/production rates are assumed to be proportional to the specific growth rate (Eq. 5) during the process but this is not always the case. Monod-type models can also be used to describe other specific consumption/production rates of metabolites independent of growth (Baughman et al. 2010; Dorka et al. 2009; Gao et al. 2007; Provost and Bastin 2004). Batt and Kompala (1989), Glacken et al. (1988), and Ozturk et al. (1992) described a four-compartment structured model to describe growth of hybridoma and monoclonal antibody productions using Monod- and Haldane-type kinetic models.

**Table 2:** Kinetic models for mammalian cell growth

Kinetic parameters	Cells	References	Growth equations
${\mu}_{\max }$ = 0.125 h^−1^ ${k}_{Gln}$= 0.8 mM ${k}_{\mathrm{Lac}}$= 8 mM ${k}_{Amm}$= 1.05 mM	Hybridoma	(Bree et al. 1988)	$\mu ={\mu}_{\max }*\frac{Gln}{k_{Gln}+Gln}*\frac{k_{\mathrm{Lac}}}{k_{\mathrm{Lac}}+\mathrm{Lac}}*\frac{k_{Amm}}{k_{Amm}+Amm}$
${\mu}_{\max }$= 0.055 h^−1^ ${\left({k}_s\right)}_0$= 26.5 $\beta$= 0.21 ${k}_{Gln}$= 0.15 mM ${k}_{Amm}$= 26 mM^2^	Hybridoma	(Glacken et al. 1988)	$\mu ={\mu}_{\max }*\frac{Ser*Gln}{\left(Ser+{\left({k}_s\right)}_0*{X}^{-\beta}\right)*\left({k}_{Gln}+Gln\right)*\left(1+\frac{Amm^2}{k_{Amm}}\right)}$
${\mu}_{\max }$= 0.053 h^−1^ ${k}_{\mathrm{Serum}}$= 0.0139 *v*/*v*	Hybridoma	(Ozturk and Palsson 1991)	$\mu ={\mu}_{\max }*\frac{\mathrm{Serum}}{k_{\mathrm{Serum}}+\mathrm{Serum}}$
${\mu}_{\max }$= 0.045 h^−1^ ${k}_{Glc}$= 1 mM ${k}_{Gln}$= 0.3 mM	Hybridoma	(de Tremblay et al. 1992)	$\mu ={\mu}_{\max }*\frac{Glc}{k_{Glc}+Glc}*\frac{Gln}{k_{Gln}+Gln}$
${\mu}_{\max }$= 0.036 h^−1^ ${k}_{Gln}$= 0.06 mM	Hybridoma	(Pörtner et al. 1996)	$\mu ={\mu}_{\max }*\frac{Gln}{k_{Gln}+Gln}$
${\mu}_{\max }$= 0.689 h^−1^ ${k}_{Glc}$= 4.79 mM ${k}_{Gln}$= 0.032 mM ${k}_{Lac}$= 0.67 mM ${k}_{\mathrm{rd}}$= 0.019 h^−1^ ${k}_A$= 0.275 h^−1^	Hybridoma	(Dhir et al. 2000)	$\mu ={\mu}_{\max }*\frac{Glc}{k_{Glc}+Glc}*\frac{Gln}{k_{Gln}+Gln}*\frac{k_{\mathrm{Lac}}}{k_{\mathrm{Lac}}+\mathrm{Lac}}-{k}_{\mathrm{rd}}\frac{\mathrm{Lac}}{k_{dLac}+\mathrm{Lac}}-{k}_A*Amm$
${\mu}_{\max }$= 0.065 h^−1^ ${k}_{Glc}$= 0.75 mM ${k}_{Gln}$= 0.075 mM ${k}_{\mathrm{Lac}}$= 90 mM ${k}_{Amm}$ = 15 mM	Hybridoma	(Jang and Barford 2000)	$\mu ={\mu}_{\max }*\frac{Glc}{k_{Glc}+Glc}*\frac{Gln}{k_{Gln}+Gln}*\frac{k_{\mathrm{Lac}}}{k_{\mathrm{Lac}}+\mathrm{Lac}}*\frac{k_{Amm}}{k_{Amm}+Amm}$
${\mu}_{\max }$= 0.028 h^−1^ ${k}_{Glc}$= 0.084 mM ${k}_{Gln}$= 0.047 mM ${k}_{\mathrm{Lac}}$= 43 mM ${k}_{Amm}$= 6.51 mM	0043HO	(Xing et al. 2010)	
${\mu}_{\max }$ = 0.0190 h^−1^ ${k}_{Glc}$= 1.45 mM	AGE1.HN	(Borchers et al. 2013)	$\mu ={\mu}_{\max }*\frac{Glc}{k_{Glc}+Glc}$*

*Glc* glucose, *Gln* glutamine, *Lac* lactate, *Amm* ammonium

*kinetic model of growth in 500mL stirred tank reactors

### Logistic equation

Verhulst (Vogels et al. 1975) developed the first logistic equation to describe population growth based on the work of Thomas Malthus. Verhulst added an extra term, *K*, called the *overall saturation constant* to the first model of Malthus to represent the resistance to growth up to a certain limit value of biomass concentration as shown in the equation describing *logistic growth.*

8 $$ \frac{dX(t)}{dt}=r\cdotp X(t)\cdotp \left(1-\frac{X(t)}{K}\right) $$

This model does not take into account the death of cells, either by necrosis or apoptosis, observed in mammalian cell processes. Therefore, cell growth and death have been taken into account in an alternative formulation, the so-called four-parameter-generalized-logistic-equation which can describe cell density profiles in batch and fed-batch cultures (Jolicoeur and Pontier 1989).

9 $$ X(t)=\frac{A}{e^{Bt}+C{e}^{-Dt}} $$

Where *X*(*t*) is the cell density at time t. *A* is related to the initial value of *X* while *B* and *C* correspond to the maximum death and growth rate, respectively. Goudar (2012a) applied such logistic modeling in batch and fed-batch cultures of mammalian cells. To describe the cell culture process system, besides Eq. 9, two types of equations were used for the formation of products and for substrate consumption.

The second type of equation used was the *logistic growth equation* to describe monotonously increasing quantities of product concentrations, *P*, such as lactate and ammonium.

10 $$ P(t)=\frac{A}{1+C{e}^{-Dt}} $$

Finally, the logistic *decline equation* has been used to describe monotonously decreasing quantities of nutrient concentration, *N*, such as glucose or glutamine concentration.

11 $$ N(t)=\frac{A}{{\mathrm{e}}^{Bt}+C} $$

To get robust logistic modeling, initial estimation of parameters using linearization has been successfully used. More complex equations can be used. For instance, Acosta et al. (2007) use two asymmetric logistic equations for growth and nutrients and products. Logistic equations have been successfully used in a variety of applications to describe the dynamic of population growth; most of them involved bacterial growth (Gibson et al. 1987; Tsoularis and Wallace 2002) but also mammalian cell growth (Goudar 2012a, b; Goudar et al. 2005, 2009). This kind of model is particularly useful if the matrix *S* from Eq. 2 is not known.

The main differences between the logistic equation and the Monod model are that the logistic equation uses fewer parameters compared to the Monod model and that it does not require knowledge about limiting substrates. That makes the computational step from logistic approach simpler than classical approaches but seems less suited for extrapolation and not well suited to incorporate additional information on metabolism.

### Neural networks and hybrid models

Neural networks (NNs) are computational models of black box type. They are used to model a wide spectrum of problems. NNs are an interconnected network structure composed of a set of processing elements (PEs) (Price and Shmulevich 2007). Giving some input, computations are made using the transfer functions of the network to estimate the output. The network is composed of different layers: the input layer, the hidden layers, and the output layer. The PEs are composed of transfer functions (polynomial, hyperbolic, kernel, …) and the significance of the connection is called the weight.

Marique et al. (2001) used a NN to simulate non-linear kinetics of CHO strains. For the transfer function, a classical sigmoid function was applied. Biomass, glucose, glutamine, lactate, and ammonia concentrations represented output and input layers. A model with CHO K1 of those five variables was obtained by using only one hidden layer. Moreover, the same NN has been used to predict the behavior of another cell line (CHO TF70R) by adjusting the time scale. As described above, mechanistic knowledge is not needed to create NNs. Nevertheless, hybrid neural networks are more used since a decade, combining non-parametric functions such as NNs and parametric functions based on cell culture process knowledge (Laursen et al. 2007; van Can et al. 1999; Vande Wouwer et al. 2004). Laursen et al. combined material balances to estimate accumulation rates of biomass, product, and metabolites in a bacterial fed-batch culture combined with a NN for each variable. Vande Wouver et al. (2004) used several hybrid NNs to describe CHO batch cultures. A set of NNs for the calculation of the reaction rates was combined with material balances of a bioreactor (Chen et al. 2000). Teixeira et al. (2007) used EFM to reduce the metabolic network of a recombinant baby hamster kidney (BHK-21A) cell line producing a glycoprotein (IgG1-IL2) to a minimal set of macroscopic reactions which then served as a basis for a hybrid NN model. A three-layered backpropagation neural network was used as a non-parametric function to describe the kinetics of the system. By using this hybrid model in a fed-batch process, they were able to increase the final productivity of IgG1-IL2 by 10 % (Teixeira et al. 2007). Graefe et al. (1999) applied a serial hybrid model to CHO-K1 by combining mass balances and neural network kinetics. A convincing prediction of components concentrations in the stirred tank bioreactor was achieved.

Hybrid models exploit the advantages of parametric models (“grey box model”) and of non-parametric models (“black box models”) and overcome the limits of each used individually. For complex problems, this kind of methodology provides a good benefit/cost ratio.

To conclude, logistic models and neural network models do not or only partially consider the underlying physical, biological phenomena. Nevertheless, they are less difficult to develop than mechanistic models. There parameters are hardly physically interpretable in contrast to mechanistic models that take into account the underlying phenomena including mass balances which supports biological understanding. Moreover, mechanistic-type models are generally more suited for extrapolation outside the experimentally explored space. For very complex systems with limited mechanistic knowledge available, logistic models and NNs can be useful due to the lower number of parameters to identify. Hybrid models take the advantages of both approaches, the mechanistic approach and the empirical/semi-empirical approach, e.g., by improving model extrapolation compared to a pure NN (Van Can et al. 1998) but require complex optimization tools to calibrate them.

## Model calibration and testing

Before starting to identify model parameters, it is important to identify and remove outliers. Outliers can increase the level of variance of the model parameters (Yang et al. 2011), can reduce the model performance by biasing parameter estimates, and can lead to false conclusion. Outliers are often due to fault, biological deviations, or human/instrumental errors. For instance, Borchers et al. (2013) defined an outlier detection approach for AGE1.HN cell line based on a model (model generic approach) by introducing an additional pessimistic bound (relative error). Then, they identified model parameters and performed a reachability analysis. The outliers were then selected by comparing the reachable state sets with the measurements data. There are many other possible methods to identify outliers like the locally estimated plot smoothing (LOESS) (Sriyudthsak et al. 2013) or splines (Laursen et al. 2007), but most of them depend on the context of the experiment, the equipment performed, and the variables analyzed.

Kinetic parameters are usually determined by fitting the model to the experimental data. Parameter estimation is an optimization problem, in which an objective or cost function characterizing the deviation of a model prediction from the experimental data is minimized by adjusting model parameters. Typically, least squares or the maximum likelihood functions are applied. Together with the usually non-linear differential equations of the model, non-convex problems result that are hard to solve, but powerful algorithms and mathematical tools have been developed to treat them. These were successfully applied to macroscopic models of mammalian cells. For instance, Borchers et al. (2013) used a semi-definite programming (SDP) algorithm to solve a polynomial function by reformulating and relaxing the non-convex constraint problem into a convex optimization problem, whereas Baughman et al. (2010) used a simple discretization scheme combined with a filtered interior point primal dual line search algorithm (IPOPT) to identify global optima for the non-convex problem.

The choice of optimization algorithms depends on the type of optimization problem, the number of parameters and variables, the constraints, the model but also software availability, e.g., gOPT from gPROMs (Kontoravdi et al. 2007), ADMIT toolbox (Borchers et al. 2013; Streif et al. 2012) and MATLAB (MathWorks, Natick, MA) (Sainz et al. 2003; Teixeira et al. 2007; Vande Wouwer et al. 2004).

Goudar et al. (2009) compared the simplex method, the generalized reduced gradient method (GRG) and the Levenberg-Marquard algorithm (LMA) for non-linear parameter estimation of logistic model parameters of batch and fed-batch mammalian cell culture. The simplex method and GRG methods resulted in a better fit than LMA. LMA was also used by Vande Wouwer et al. (2004) for batch CHO cell culture. LMA was applied in the training process for hybrid models of bioprocesses (Graefe et al. 1999). Dorka et al. (2009) successfully identified Monod-type parameters of hybridoma cell culture during exponential phase by using quadratic programming (QP). For the post-exponential phase, the maximal rates and the half-saturation constants were calibrated using a Markov chain Monte Carlo method (MCMC) using a Metropolis-Hasting algorithm. More examples of applications of optimization algorithms for macroscopic modeling of mammalian cells can be found in a number of studies, e.g., the method of Powell (Glacken et al. 1988), MCMC (Xing et al. 2010), linear programming (Sainz et al. 2003), the particle swarm algorithm (PSO) (Selişteanu et al. 2015), sequential quadratic programming (SQP) (Kontoravdi et al. 2007), and a quasi-Newton method (Teixeira et al. 2007). It is not possible to a priori recommend any single algorithm as the superior method as it is problem-dependent.

Major problems of parameter estimation in non-linear systems are the potential existence of multiple local minima and over fitting. Additionally, models have to be assessed for their predictive power and their robustness against perturbations.

Model validation is one of the most critical parts of the modeling process. We can identify two ways to evaluate the quality of a model: one called direct validation compares the model prediction with the same experimental data as used to estimate the parameters (Goudar et al. 2009; Selişteanu et al. 2015). The second method uses an independent new data set to validate or invalidate the model (cross validation). For instance, Xing et al. (2010) identified the parameters on three independent sampling trains with different initial parameters and then used two types of validation. The first one validated the model by applying the model to different cell cultures to assess the applicability of the model. Secondly, they applied the model to a perturbed system to assess the accuracy of the model. For hybrid neural models, two data sets, one training/calibration data set to identify hybrid model parameters and one validation data set to assess the model quality are usually used (Oliveira 2004; Teixeira et al. 2007; Vande Wouwer et al. 2004).

A more complex methodology was used by Borchers et al. (2013) for the invalidation of models and for parameter estimation. Their set-based method builds on a semi-definite programming relaxation and outer-bounding techniques supported by the ADMIT toolbox (Streif et al. 2012).

Another important method to assess the quality of a model is to perform sensitivity analysis that can provide valuable information regarding the importance of parameters on the model output and on the possible impact of variability of the input on the output. For instance, one can evaluate the largest possible variation of the parameters which does not lead to rejection of the model (Borchers et al. 2013). Baughman et al. (2010) quantified the impact of the linear discretization on the parameters and on the numerical error. Moreover, the impact of possible measurement variability on model estimate has been performed by using Monte Carlo simulation using normal distribution (Baughman et al. 2010). Global sensitivity analysis (GSA) has the advantage of evaluating the effect of a factor while all other factors varied simultaneously (Kiparissides et al. 2008). For instance, Kontoravdi et al. (2007) used the Sobol’ global sensitivity method to assess the sensitivity of the parameters of dynamic hybridoma model and, based on the same case study, Kiparissides et al. (2008) evaluated the performance of the Sobol’ method and derivative based global sensitivity measures (DGSM) as a GSA method. The DSGM method was identified as more useful than the Sobol’ method due to the lower computational requirement while producing the same quality of results.

## Application of models for control of processes

An important application for industrial production is the use of macroscopic models for the control of production processes. Generally, models applied for control should be simple and variations of process conditions, and cell characteristics can be taken into account by adapting the model parameters online. It is most straightforward to use a stoichiometric model together with dynamic material balances to estimate the state of a culture. A feeding strategy can be determined in a fed-batch process based on the model and on defining an objective to reach. For instance, Haas et al. (2001) used an open-loop-feedback-optimal controller to maintain glucose and glutamine at low levels in a culture of a hybridoma cell line producing an IgG antibody. This controller was based on a Monod-type model of growth in which the parameters and the state are estimated, and then an optimization part calculates an optimal feeding profile. Teixeira et al. (2007) also used a controller with a hybrid model on a culture of a BKH cell line producing an IgG1-IL2. The glucose and glutamine feeding rate was optimized to maximize the total amount of antibody produced at the end of the experiment. Finally, Craven et al. (2014) used a non-linear model predictive controller (NMPC) in a CHO cell line to control the glucose concentration. The kinetic models used were of Monod type.

## Conclusion and outlook

As was described in this review, setting up of macroscopic models is carried out in primarily two steps (Fig. 1). After identification of the stoichiometric part of a model, kinetics for growth and metabolite conversion are defined to yield relatively simple yet useful combined models. As in most other cases of modeling, macroscopic modeling of mammalian cell cultures is an iterative process of setting up a model, calibrating, validating, and testing it; designing and performing new experiments; and revising the model.

Macroscopic modeling of metabolism can be used in many applications to accelerate cell line selection, medium optimization, feeding strategy development, and other bioprocess development activities. By using macroscopic models, it is possible to understand what are the significant parameters that have an impact on the cell culture process and then predict how the process will evolve if one parameter is changed. Having predictive models of cell culture processes can be a powerful tool to help in identifying the critical process parameters which have an impact on CQAs, e.g., glycosylation, and to optimize process performance with respect to a defined objective function. Therefore, macroscopic models are more and more used by biopharmaceutical companies. They also assist in fulfilling requirements of the QbD methodology by providing a handle to further improve CQAs.

For fed-batch cell culture processes, macroscopic models can be applied to predict the time courses of metabolites which have significant impact on the cell culture process or to estimate process rates of interest and then control feeding rates based on model prediction and using an appropriate objective function.

Different types of models can be used to select variables and determine the macroscopic reactions of the system, and then, different kinetic models can be applied to simulate and predict the macroscopic behavior of the cells. All types of combinations of those models can be applied; for example, a stoichiometric model using the EFM method combined with a logistic kinetic equation or empirical stoichiometric relationships identified with a PCA combined with Monod-type kinetic equations and so on. Stoichiometry derived by the EFM method are used together with NNs to result in so-called hybrid neural network models. Such kind of hybrid models are more of grey box type rather than black box type (Laursen et al. 2007; van Can et al. 1999; Vande Wouwer et al. 2004). The choice of the model depends not only on the aim of the study but also on the complexity of the system we want to simulate and understand. As summary of strategies used to develop macroscopic models with mammalian cells from various studies is presented on Table 3.

**Table 3:** Applied strategies to develop macroscopic models for mammalian cells

Input-output relationship methodology	Kinetic model		Model variables			Parameter estimation		Cell line	Reference
–	Logistic		Cell density NH_3_ Lactate	Glucose Gln		LMA Simplex GRG		CHO BHK Hybridoma	(Goudar et al. 2009)
Metabolic network	Hybrid model (serial and parallel)		Cell density Lactate	Glucose Gln		LMA		CHO	(Vande Wouwer et al. 2004)
Metabolic network + EFM	Monod type		Cell density NH_3_ Lactate Gln	Asp Asn Pro Mab		Quadratic programing (exponential phase)	MCMC method (post-exponential phase)	Hybridoma	(Gao et al. 2007)
Expert reasoning	Monod type Inhibition type		Cell density Glucose CO_2_	Gln Lactate NH_3_ Mab		Literature data		Hybridoma	(Batt and Kompala 1989; Glacken et al. 1988; Ozturk et al. 1992)
Expert reasoning	Monod type		Cell density Gln	NH_3_ Mab		Powel method		Hybridoma CRL-1606	(Glacken et al. 1988)
Expert reasoning	Monod type	First principle	Cell density Glucose Gln	Lactate NH_3_ Mab		MCMC method		CHO	(Xing et al. 2010)
PFA	Monod type Canonical		Cell density Glucose CO_2_	Gln Lactate NH_3_ Mab		Monte Carlo simulation and Canonical algorithm		CHO	(Xing et al. 2008)
Metabolic network	First principle		Cell density Glucose	NH_3_ Ethanol Glycerol		Linear programming		Yeast	(Sainz et al. 2003)
Expert reasoning	Hybrid serial		Cell density Glucose		Ethanol	Large-scale SQP		Baker’s yeast	(Oliveira 2004)
Expert reasoning (from Gao et al.)	Monod type		Cell density NH_3_ Lactate Gln	Asp Asn Pro Mab		PSO		Hybridoma 130-8F	(Selişteanu et al. 2015)
Metabolic network + EFM	Hybrid model		Cell density Glucose Gln	Lactate NH3 Ala Mab		Quasi-Newton algorithm with conjugate gradient line search		BHK-21A	(Teixeira et al. 2007)
Expert reasoning	First principle		Cell density Glucose Gln	Lactate NH_3_		SDP		AGE1.HN	(Borchers et al. 2013)
Expert reasoning	Monod type	First principle	Cell density Glucose Gln Lactate	NH_3_ Mab Glycosylation		SQP		Hybridoma 14-4-4S	(Kontoravdi et al. 2007)
Expert reasoning (from Gao et al.)	Monod type		Cell density NH_3_ Lactate Gln	Asp Asn Pro Mab		IPOPT		Hybridoma 130-8F	(Baughman et al. 2010)

### Ethical statement

This article does not contain any studies with human participants or animals performed by any of the authors.
